# Risk factors associated with unplanned readmissions and frequent out-of-hour emergency department visits after pediatric tracheostomy: a nationwide inpatient database study in Japan

**DOI:** 10.1007/s00431-025-06242-1

**Published:** 2025-06-17

**Authors:** Ai Ito-Shinjo, Daisuke Shinjo, Tomoo Nakamura, Mitsuru Kubota, Kiyohide Fushimi

**Affiliations:** 1 Department of General Pediatrics and Interdisciplinary Medicine, National Center for Child Health and Development, Tokyo, Japan; 2 Department of Health Policy and Informatics, Graduate School of Medical and Dental Sciences, Institute of Science Tokyo, Tokyo, Japan

**Keywords:** Tracheostomy, Readmission, Emergency department visits, Complications, Health outcomes

## Abstract

Children who undergo tracheostomy usually experience unexpected readmissions and frequent emergency department (ED) visits. Therefore, identifying the risk factors associated with medical resource use may help improve health outcomes. This study aimed to describe the clinical features of children who underwent tracheostomy and determine the risk factors for unplanned readmission and frequent out-of-hour ED visits. Data of children aged between 0 and 18 years who underwent tracheostomy and were discharged between April 2016 and September 2018 were retrieved from the Japanese National Inpatient Database. Risk factors for readmission and frequent out-of-hour emergency department visits within 180 days of tracheostomy were identified using multivariate logistic regression analysis. Overall, 1112 patients underwent tracheostomy during the study period; 483 (43%) patients were readmitted, and 220 (20%) visited the ED frequently. The multivariate logistic regression analysis showed that < 1 year of age (odds ratio (OR) = 1.77; 95% confidence interval (CI) 1.26–2.47; *p* < 0.05), tube feeding (OR = 1.36; 95% CI 1.03–1.80; *p* < 0.05), neurological impairment (OR = 1.52; 95% CI 1.02–2.25; *p* < 0.05), and mechanical ventilation (OR = 1.43; 95% CI 1.03–1.99; *p* < 0.05) were risk factors for readmissions. In contrast, < 1 year of age (OR = 1.53; 95% CI 1.03–2.27; *p* < 0.05), home oxygen therapy (OR = 1.94; 95% CI 1.29–2.92; *p* < 0.05), and unplanned tracheostomy (OR = 2.38; 95% CI 1.05–5.40; *p* < 0.05) were risk factors for ED visits.

*Conclusions*: This study describes the clinical features and risk factors for readmission and frequent out-of-hour ED visits after tracheostomy. The findings may contribute to improving health outcomes, healthcare plans, and evidence-based policymaking.

**Table:** 

**What Is Known:** *• The use of tracheostomy in children with medical complexity has increased, and these children are known to be frequent users of medical resources.* *• Multicenter studies dealing with the long-term outcomes of pediatric tracheostomy and risk factors associated with high medical resource use are still limited.* **What Is New:** *• Using the nationwide database in Japan, this study identified several risk factors associated with unplanned readmissions and out-of-hours emergency department visits (less than 1 years of ages, dependent on medical technologies, etc.).* *• This study contributes for coordinating appropriate home care plans and reducing preventable medical resource use.*

**Supplementary Information:**

The online version contains supplementary material available at 10.1007/s00431-025-06242-1.

## Introduction

Tracheostomy is performed in patients with upper airway obstruction or respiratory failure requiring long-term ventilator support [1, 2]. The use of tracheostomies has been increasing, particularly among children with medical complexity (CMC), including those with neurological impairment (NI) who require home ventilator support [3–5].

A previous study reported that hospitalized CMCs accounted for < 1% of the population; however, they incurred one-third of the medical costs associated with pediatric hospitalizations [6]. Recently, the understanding of these high medical users and improvement of care coordination has grown due to their potential to improve outcomes and reduce unnecessary medical healthcare utilization [7].

Among CMCs who have severe and complex conditions or require ventilators, many frequently visit the emergency department (ED) and are readmitted shortly after undergoing tracheostomies [8, 9]. These reports indicate that children who undergo tracheostomy require frequent use of medical resources. They are commonly hospitalized for various reasons, including pneumonia (both typical respiratory infections and aspiration pneumonia) and tracheostomy-related complications.

Although the American Academy of Otolaryngology-Head and Neck Surgery Foundation recognizes the importance of investigating risk factors for readmission and ED visits after tracheostomy [7], the available evidence remains limited. Only a few multicenter studies have addressed the outcomes of tracheostomy [8–11], and a limited number of reports have investigated the risk factors for readmissions and frequent long-term ED visits after tracheostomy [4, 8, 9]. Consequently, information on a large cohort of multicenter epidemiological data and the long-term outcomes of children who underwent tracheostomy in Japan is limited [12]. Identifying the clinical features and risk factors associated with medical resource use in children is beneficial for improving health outcomes and healthcare plans [13, 14].

Therefore, this study aimed to describe the clinical characteristics of children who underwent tracheostomy in Japan and to identify the risk factors for unplanned readmissions and frequent out-of-hour emergency department (ED) visits following their tracheostomy hospitalization, using the Japanese administrative database.

## Material and methods

This retrospective study’s cohort was identified using the Japanese Administrative Database, Diagnosis Procedure Combination/per-diem payment system (DPC/PDPS). Specifically, this database is a case-mix patient classification system, which has been linked to payments at acute care hospitals in Japan since 2003. Details of this database have been described previously [15, 16]. The DPC/PDPS covered 1730 hospitals and 488,563 beds in 2018, and almost all acute inpatients were included, accounting for more than half of the 894,000 hospital beds in Japan [15, 17].

Patient clinical and administrative claims data were collected annually from hospitals. This database included information on age; sex; diagnostic information using the International Statistical Classification of Diseases, Tenth Revision (ICD-10) code; all procedures; medical device use; medications; the purpose of admission; types of admission (planned or unplanned); duration of admission; outcome at discharge; and discharge destination. It also included hospital information regarding the number of beds for pediatric patients.

This study was approved by the Institutional Review Board of the Institute of Science Tokyo (M2024-075). The board determined that informed consent was not required because the data were anonymized.

To avoid the impact of the coronavirus disease 2019 (COVID-19) pandemic, we included children aged 0 to 18 years who underwent tracheostomy (Japanese operative code: K386-00) and were discharged from Japanese hospitals between April 2016 and September 2018. Data from October 2018 to March 2019 were used for follow-up. Index hospitalizations were defined as those in which the patient underwent tracheostomy for the first time. Patients were excluded if they died during the index hospitalization, underwent tracheostomy closure (K396-00), were transferred to other hospitals or post-acute care facilities, or were scheduled for transfer to other hospitals during the index hospitalization.

The primary outcome was unplanned readmission for treatment within the same hospital during the first 180 days after the index hospitalization in patients who had undergone a tracheostomy. While the follow-up period of studies on prognostic prediction after surgery commonly employed 30 days or less [1, 16, 18], as a large number of CMCs were expected in our cohort, we followed up for a longer period according to previous studies [8–11]. We set the follow-up period at 180 days considering due to the characteristics of DPC database as well. Because it can only track visits to the same hospital, as the observation period lengthens, an increasing number of patients visit other hospitals, making it difficult to accurately assess the visits and admissions. We excluded planned readmission, which usually included hospitalizations for respite care, and only considered readmissions for treatment since CMCs sometimes use respite admissions after significant changes, such as starting mechanical ventilation in Japan. Generally, complex cases are associated with background diseases and various conditions, leading to longer hospital stays and more visits and readmissions. Furthermore, the secondary outcome was the frequency of ED visits within the same hospital during the first 180 days after the index hospitalization. Frequent out-of-hour ED visits were defined as two or more night/holiday ED visits according to previous reports [11, 19, 20].

Individual- and hospital-level data were obtained from the DPC and PDPS. Individual data included age, sex, ICD-10 diagnosis, procedures, medical device use at discharge, length of hospital stay, admission status (planned or unplanned), purpose of admission, outcome at discharge, discharge destination, and the distance between the hospital and the patient’s home.

Comorbidities were categorized into the following groups according to previous studies: upper airway anomaly, NI, prematurity, trauma, and others [10, 21–23]. Upper airway anomaly, NI, prematurity, and trauma were not mutually exclusive. Cases that did not possess an ICD-10 code for those four groups were classified as “others” (Online Resource 1). Respiratory support was categorized according to the degree of support as follows: non-respiratory support, only home oxygen therapy (HOT) use, and home mechanical ventilation support (with or without HOT). Tube nutrition use included children who used all types of tube feeding, with or without gastrostomy. The diagnosis at readmission was categorized into the following groups according to previous studies using ICD-10 code: respiratory, digestive, nervous system, infectious, and others [10, 11].

We used the Manhattan distance to measure the distance between the hospital and the patient’s home. However, as some samples lacked Manhattan distance data, we recruited straight-distance data. The indicator for bed size in the pediatric ward was categorized into the lowest quartile (< 1652 pediatric patients/year), interquartile (1652–3137 pediatric patients/year), and highest quartile (≥ 3137 pediatric patients/year). Furthermore, the clinical course classification before tracheostomy was separated into three categories as follows: planned tracheostomy (planned admission and receiving tracheostomy within 3 days post-admission), tracheostomy during hospitalization postpartum (admission within 0–7 days of age), and unplanned tracheostomy (patients ineligible for the other two categories). Unplanned tracheostomy is distinct from emergency tracheostomy.

Continuous variables are presented as medians and interquartile ranges (IQRs) or means ± standard deviations (SDs), depending on their distribution. Pearson’s chi-square and Kruskal–Wallis’s tests were used to analyze the comparisons between the readmission and non-readmission groups and between the groups with and without frequent after-hour visits. Multivariate logistic regression analysis was used to estimate predictors associated with the 180-day unscheduled hospital readmissions and after-hour ED visits. The results are presented as odds ratios (ORs) and 95% confidence intervals (CIs). All analyses were two-tailed, and statistical significance was set at *p* < 0.05. Multicollinearity between covariates was determined using the variance inflation factor and tolerance values. All analyses were performed using EZR version 1.54.33.

## Results

### Participant characteristics

**Fig. 1:**
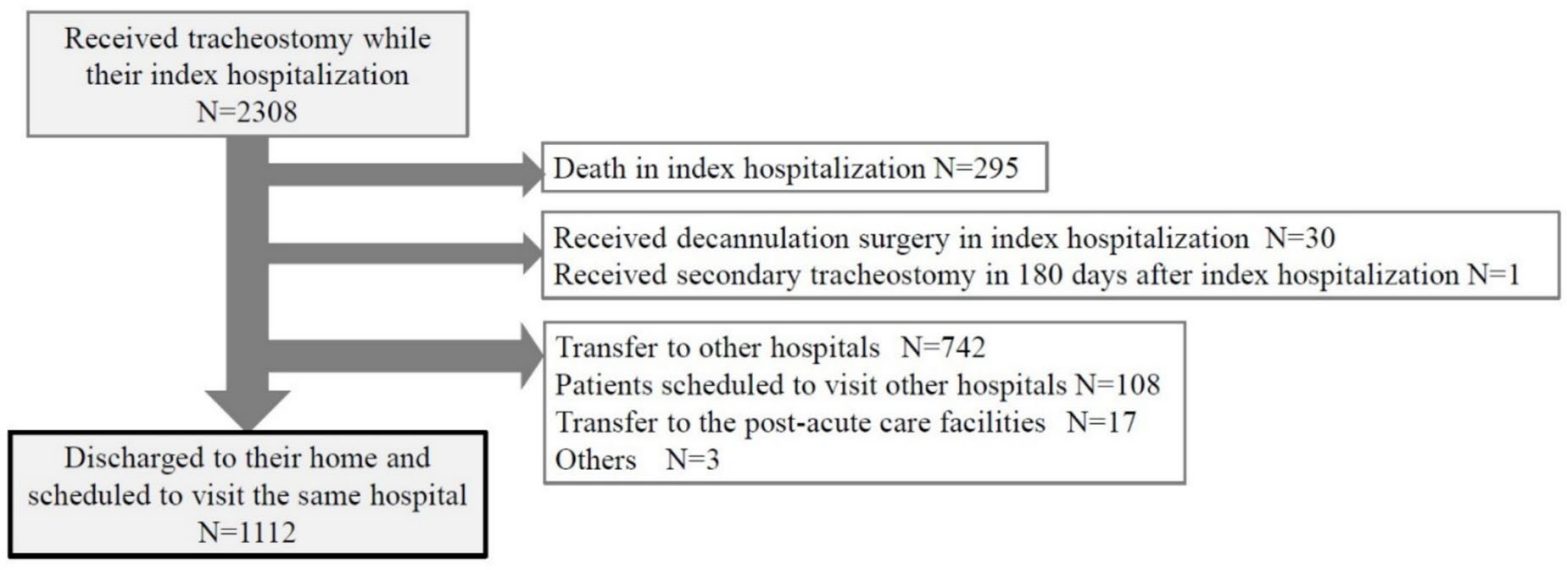
Construction of the pediatric cohort that underwent tracheostomy

A total of 2,308 patients underwent tracheostomy during their index hospitalization across 219 hospitals (Fig. 1). After excluding patients who met the exclusion criteria, 1112 patients were discharged to their homes and scheduled to visit the same hospital. No cases were excluded due to lack of data. Additionally, no significant differences were found in characteristics between the study cohort and excluded patients. Table 1 presents the characteristics of the study cohort. The median age at index hospitalization was 0 (IQR 0–6) years, and 601 patients (54%) were aged < 1 year. NI (69%) was the most common comorbidity associated with tracheostomy, followed by upper airway anomalies (21%) and prematurity (18%). More than 60% of patients depended on respiratory support, and 45% used feeding tubes at discharge from their index discharge. Unplanned tracheostomy was performed in 63% of cases.

**Table 1:** Characteristics and variables associated with readmission and frequent out-of-hour ED visits within 180 days of tracheostomy according to groups

Variables	Total *N* = 1112	Readmission *N* = 374	Non-readmission *N* = 738	*p*-value	≥ 2 visits *N* = 220	< 2 visits *N* = 892	*p*-value
Age, years, median (IQR)	0 (0–6)	0 (0–3.0)	0 (0–8.0)	< 0.05	0 (0–2.0)	0 (0–8.0)	< 0.05
< 1 year of age, *n* (%)	601 (54)	231 (62)	370 (50)	< 0.05	140 (64)	461 (52)	< 0.05
Male, *n* (%)	596 (54)	196 (52)	400 (54)	0.61	118 (54)	478 (54)	1.00
Comorbidities associated with tracheotomy^*a*^, *n* (%)							
A. Upper airway anomaly	233 (21)	76 (20)	157 (21)	0.76	60 (27)	241 (27)	0.93
B. Neurological impairment	762 (69)	284 (76)	478 (65)	< 0.05	156 (71)	606 (68)	0.42
C. Prematurity	200 (18)	63 (17)	137 (19)	0.51	43 (20)	157 (18)	0.49
D. Trauma	42 (4)	5 (1)	37 (5)	< 0.05	4 (1.8)	38 (4.3)	0.11
E. Others	163 (15)	43 (12)	120 (16)	< 0.05	28 (13)	135 (15)	0.30
Respiratory support at discharge, *n* (%)				< 0.05			< 0.05
None	432 (39)	110 (29)	322 (44)		63 (29)	369 (41)	
Only home oxygen therapy	259 (23)	167 (23)	92 (25)		71 (33)	188 (21)	
Ventilation support	421 (38)	172 (46)	249 (34)		86 (39)	335 (38)	
Tube nutrition at discharge, *n* (%)	495 (45)	202 (54)	293 (40)	< 0.05	110 (50)	385 (43)	0.07
Length of stay of initial admission, days, median (IQR)	113 (58–221)	133 (64–240)	107 (53–212)	< 0.05	112 (56–213)	113 (58–222)	0.93
Number of pediatric admissions^b^, *n* (%)				0.82			0.10
< 1652/year	279 (25)	95 (25)	184 (25)		46 (21)	233 (26)	
1652–3137/year	568 (51)	185 (50)	383 (52)		111 (51)	457 (51)	
≥ 3137/year	265 (24)	94 (25)	171 (23)		63 (29)	202 (23)	
Distance between home and hospital, *n* (%)				< 0.05			< 0.05
< 4.9 km	276 (25)	108 (29)	168 (23)		61 (28)	215 (24)	
4.9–20.7 km	553 (51)	190 (51)	363 (49)		129 (59)	424 (48)	
≥ 20.7 km	275 (25)	74 (20)	201 (27)		30 (14)	245 (28)	
N/A	8 (1)	2 (1)	6 (1)		0 (0)	8 (1)	
Clinical course classification^c^, *n* (%)				0.11			< 0.05
Planned tracheostomy	80 (7)	61 (8)	19 (5)		7 (3)	73 (8)	
Tracheostomy during hospitalization postpartum	333 (30)	120 (32)	213 (29)		76 (35)	265 (30)	
Unplanned tracheostomy	699 (63)	235 (63)	464 (63)		137 (62)	552 (62)	

*N/A* not applicable, *IQR* interquartile range, *ED* emergency department. *p* < 0.05

^*a*^Upper airway anomaly, neurological impairment, prematurity, and trauma were not mutually exclusive. Cases that did not possess an ICD-10 code for those four groups were classified as “others”

^*b*^The number of pediatric admissions was calculated using the number of pediatric patients per year as an indicator of the bed size of the ward for children

^*c*^Planned admission and receiving tracheostomy within 3 days after admission was defined as “planned tracheostomy.” Cases admitted within 0–7 days of age at index hospitalization were defined as “tracheostomy during hospitalization postpartum.” Those that did not fall into the two groups were categorized as “unplanned tracheostomy”

### Unplanned readmissions within 180 days of tracheostomy

Among the 1112 patients, 374 (34%) were readmitted within 180 days of tracheostomy (Table 1). Children readmitted within 30 days were the most common group (172/374 patients). Specifically, 15%, 25%, and 32% were readmitted within 30, 90, and 150 days of tracheostomy, respectively. The readmission group was younger than the non-readmission group (*p* < 0.05); being < 1 year of age was more common in the readmission group than the non-readmission group (62% vs. 50%, respectively; *p* < 0.05). Tube nutrition at discharge was more common in the readmission group than in the non-readmission group (*p* < 0.05). Among the comorbidities, trauma was less common in the readmission group than in the non-readmission group (1% vs. 5%, respectively; *p* < 0.05). A respiratory disorder (54%) was the most common reason for readmission, followed by a nervous system disorder (20%) (Table 2).

**Table 2:** Clinical features of patients with readmissions within 180 days of tracheostomy

Clinical features	Total *N* = 374
Length of stay of readmissions, days, median (IQR)	9.0 (5.0–16.0)
Reason for readmissions, *n* (%)	
Respiratory	246 (66)
Digestive	10 (3)
Nervous system	28 (7)
Infectious	20 (5)
Others	70 (19)

*IQR* interquartile range

**Fig. 2:**
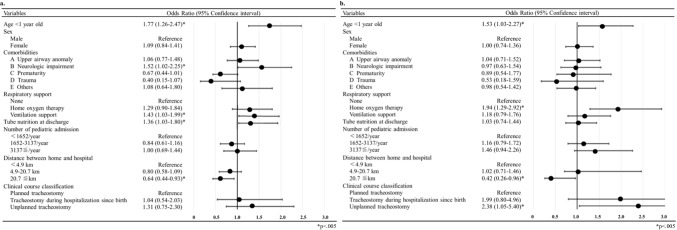
Multivariate regression analysis of risk factors for readmissions **a** and out-of-hour emergency department visits **b** within 180 days of tracheostomy

Figure 2a shows the multivariate logistic regression analysis of the risk factors associated with readmission within 180 days after tracheostomy. Notably, < 1 year of age (OR = 1.77; 95% CI 1.26–2.47; *p* < 0.05), tube feeding (OR = 1.36; 95% CI 1.03–1.80; *p* < 0.05), NI (OR = 1.52; 95% CI 1.02–2.25; *p* < 0.05), and ventilation support (OR = 1.43; 95% CI 1.03–1.99; *p* < 0.05) were risk factors for readmission within 180 days after tracheostomy.

### Frequent out-of-hour ED visits within 180 days of tracheostomy

Among the 1112 patients, 220 (20%) made frequent out-of-hour ED visits within 180 days of tracheostomy (Table 1). This group was younger than the group without frequent ED visits (*p* < 0.05). Specifically, being < 1 year of age was more common in this group than in the group without frequent ED visits (64% vs. 52%, respectively; *p* < 0.05). Multivariate logistic regression analysis (Fig. 2b) showed that < 1 year of age (OR = 1.53; 95% CI 1.03–2.27; *p* < 0.05), HOT (OR = 1.94; 95% CI 1.29–2.92; *p* < 0.05), a hospital distance > 20.7 km (OR = 0.42; 95% CI 0.26–0.96), and unplanned tracheostomy (OR = 2.38; 95% CI 1.05–5.40; *p* < 0.05) were associated with out-of-hour ED visits within 180 days of tracheostomy.

## Discussion

To the best of our knowledge, this is the first nationwide study in Japan on unplanned readmission and out-of-hour ED visits among children who underwent tracheostomy. This study adds to previous studies by analyzing risk factors for readmissions and out-of-hour ED visits, considering factors related to patient characteristics and those that may affect medical resource use, such as differences between hospital bed size, distance to hospitals, and type of clinical course.

We detected 2308 children who underwent tracheostomy in our database. According to all Japan open data on receipt information known as the “National Database of Health Insurance Claims and Specific Health Checkups of Japan Open Data [24]” categorized by surgical procedure, 2564 cases occurred in the 0–19-year-old age group (474 cases in the 15–19-year-old age group) between April 2016 and March 2019. Although the age classifications differ slightly, our database is estimated to cover over 90% of pediatric tracheostomy in Japan.

Additionally, 20% of patients were readmitted within 90 days after tracheostomy, and > 40% were readmitted within 180 days after index hospitalization. While most previous studies were conducted over a short period, our results highlight the importance of long-term follow-up for children who undergo tracheostomy. The characteristics of our study cohort were similar to those in previous reports, with half of the cases being < year of age, approximately 40% on mechanical ventilators and tube feeding, and about 70% having NIs. Surprisingly, over 60% of the tracheostomies were unplanned, which has not been reported previously. Therefore, appropriate assessment of tracheostomy and daily care in cases of deteriorating health conditions is important, and guidelines on the indications for pediatric tracheostomy in chronic conditions are required.

Respiratory-related diseases were the most common cause of readmission, consistent with previous studies where respiratory-related diseases, such as respiratory failure and airway infection, were the most common causes of readmissions [4, 8, 9]. This finding indicates the importance of expectoration, education of care, and prevention of respiratory infection among CMCs.

Comparing our study with previous research, the readmission rates in Japan were lower than those in other developed countries: 30 days (17% vs. 18–45%, respectively) [1, 8, 24], 90 days (25% vs. 44%, respectively) [4], and 180 days (34% vs. 63–66%, respectively) [9, 25] after tracheostomy. This is partly because this study was limited to readmissions to the same hospital and those for treatment, which may have underestimated hospitalization rates. Differences in the indications for tracheostomy, discharge criteria, and healthcare systems may have also influenced this disparity. Most studies did not consider avoidable or unavoidable readmissions. Therefore, further studies on unavoidable readmissions and long-term outcomes are required due to the internationally high readmission rates from long-term assessments.

In this study, < 1 year of age, NI, tube feeding, and ventilation support were identified as risk factors for readmission within 180 days after tracheostomy. Infants and mechanical ventilators have been identified as risk factors for readmission and frequent medical resource use in previous reports [9, 21, 26]. Younger patients, especially those < 1 year of age, experienced increased medical complexity with a higher risk of mortality and adverse events [10]. Additionally, infants are known to be immunologically vulnerable, and their caregivers, who just started raising their child, are usually unfamiliar with care; even if they have no medical technologies, they are potentially at risk of using hospital resources. Complex cases, such as those with NIs and children depending on medical technology, are known to use a large proportion of medical resources [6, 8]. Therefore, preparing sufficient care training and dense care plans, such as home-visiting nurses or doctor plans, before initial discharge for these children is advisable [27, 28].

In our cohort, 220 (20%) patients visited the ED within 180 days of tracheostomy. Frequent ED visits have been associated with readmission [20]. The risk factors tended to be similar to those for readmission, although children using only HOT had a higher risk of ED visits than those on mechanical ventilation, and patients living farther from the hospital had a lower risk of ED visits. Patients living long distances from hospitals usually visit multiple hospitals [19]. Since we only tracked visits to the same hospital, a potential bias exists in this study, and the frequency of visits may have been underestimated, particularly among the group with a hospital distance of > 20.7 km. Additionally, home ventilators may not be a risk factor for ED visits since severely complex cases, such as children depending on home ventilators, are typically supported by home-visiting doctors and nurses in Japan.

We found that unplanned tracheostomy was associated with a higher risk of frequent out-of-hour ED visits than planned cases. While guidelines for managing pediatric patients undergoing tracheostomy in the acute care setting exist [29], no evidence currently links the clinical course before tracheostomy to hospital resource use. Therefore, further research is needed to identify why unplanned tracheostomies are performed and why ED visits are frequent, which may reveal important factors in addressing this issue. Tracheostomy after appropriate evaluation during non-emergency conditions reduces the risk of frequent out-of-hour ED visits in children with unstable airways.

Furthermore, < 1 year of age was a common risk factor, and children depending on medical technologies have some risk of medical resource use. Emergency visits and subsequent hospital admissions have been reported to increase the length of hospital stay and lead to increased costs [30]. Therefore, providing close care planning and patient education for children with these risk factors to minimize the use of medical resources is important, not only to improve patients’ health outcomes and quality of life but also to address the issue of social costs. The care of children with medical technology, such as tracheostomy independence, places a heavy burden on families [31]. Consequently, further research into the needs of the family is required, as it is necessary to secure personnel and improve the uneven distribution of home medical care in order to expand the welfare system and services to ensure that the burden of care does not fall solely on the family.

This study had several limitations. First, the characteristics of this database imposed certain constraints on the level of detail and accuracy of the data. We could only track readmissions and visits to the same hospital. However, CMCs are generally less likely to be transferred to other hospitals; therefore, we focused on patients who planned on attending the outpatient department of the hospital in which they were admitted for their index hospitalization. This is related to the reason for the follow-up period, 180 days; more patients tend to visit other hospitals as the observation period lengthens. Although we selected unplanned admissions among readmissions within 30 days after tracheostomy, we adopted admissions for treatments in the absence of the same information between 31 and 90 days after the index discharge, which may have included a small number of hospitalizations for respite. Our database system did not capture daytime ED visits, and we could not investigate the reasons for these visits. In addition, we could not obtain detailed information on the characteristics of the home ventilator and family background environment (structure, income, and educational standards of their parents). Second, disease classification of comorbidities was based on previous reports since no validated classification of diseases exists. Third, there may be differences in ICD-10 coding between hospitals. Differences in billing reimbursement might also affect the choice of codes for a patient during hospitalization. Finally, regional differences in the density of support by home-visiting doctors and nurses may have been associated with readmissions; however, we could not account for this due to a lack of detailed data on home care medicine.

In conclusion, we found that 43% of children required unplanned readmission after tracheostomy, and 20% experienced frequent out-of-hour ED visits within 180 days after tracheostomy. We identified age < 1 year, tube feeding, NI, and ventilation support as risk factors for readmission. Age < 1 year, HOT, a hospital distance of > 20.7 km, and unplanned tracheostomy as risk factors for out-of-hour ED visits within 180 days of tracheostomy. Therefore, estimating these risk factors before the index discharge would be helpful in coordinating appropriate home care plans and reducing preventable medical resource use. This study may help improve health outcomes, healthcare plans, and evidence-based policymaking. However, further research taking into account for additional factors may be required to validate our findings.

## Supplementary Information

Below is the link to the electronic supplementary material.

Supplementary file1 (DOCX 18.2 KB)

## Data Availability

Data cannot be disclosed to the public due to a license agreement and ethical issues in each participating facility. To request the dataset generated during this study, please contact the Office of Life Science and Bioethics Research Center via: Email:infobec@tmd.ac.jp Telephone: + 81–3-3813–6111. The corresponding author is also available for data requests.

## Abbreviations

CIs: Confidence intervals
CMC: Children with medical complexity
ED: Emergency department
HOT: Home oxygen therapy
IQRs: Interquartile ranges
NI: Neurological impairment
ORs: Odds ratios
SDs: Standard deviations
