# Correction to: Risk factors associated with unplanned readmissions and frequent out-of-hour emergency department visits after pediatric tracheostomy: a nationwide inpatient database study in Japan

**DOI:** 10.1007/s00431-025-06359-3

**Published:** 2025-08-08

**Authors:** Ai Ito-Shinjo, Daisuke Shinjo, Tomoo Nakamura, Mitsuru Kubota, Kiyohide Fushimi

**Affiliations:** 1 Department of General Pediatrics and Interdisciplinary Medicine, National Center for Child Health and Development, Tokyo, Japan; 2 Department of Health Policy and Informatics, Graduate School of Medical and Dental Sciences, Institute of Science Tokyo, Tokyo, Japan

**Correction to: European Journal of Pediatrics (2025) 184:422**

10.1007/s00431-025-06242-1

This erratum corrects errors in the original publication.

**Corrections:**

**Correction 1 (Abstract):**

- **Original:** "Data of children aged between 0 and 18 years who underwent tracheostomy and were discharged between April 2016 and March 2019 were retrieved from the Japanese National Inpatient Database and retrospectively analyzed. Risk factors for readmission and frequent out-of-hour ED visits within 180 days of tracheostomy were identified using multivariate logistic regression analysis."
- **Revised:** "Data of children aged between 0 and 18 years who underwent tracheostomy and were discharged between April 2016 and September 2018 were retrieved from the Japanese National Inpatient Database. Risk factors for readmission and frequent out-of-hour emergency department visits within 180 days of tracheostomy were identified using multivariate logistic regression analysis."

**Correction 2 (Material and Methods):**

- **Original:** "We recruited children aged between 0 and 18 years who underwent tracheostomy (Japanese operative code: K386-00) and were discharged from Japanese hospitals between April 2016 and March 2019 to avoid the effect of the coronavirus disease 2019 pandemic. Hospitalizations in which the patient underwent a tracheostomy for the first time were defined as index hospitalizations. Patients who died during the index hospitalization, underwent tracheostomy closure (K396-00) during the index hospitalization, were transferred to other hospitals and post-acute care facilities, or were scheduled to visit other hospitals were excluded."
- **Revised:** "To avoid the impact of the coronavirus disease 2019 (COVID-19) pandemic, we included children aged 0 to 18 years who underwent tracheostomy (Japanese operative code: K386-00) and were discharged from Japanese hospitals between April 2016 and September 2018. Data from October 2018 to March 2019 were used for follow-up. Index hospitalizations were defined as those in which the patient underwent tracheostomy for the first time. Patients were excluded if they died during the index hospitalization, underwent tracheostomy closure (K396-00), were transferred to other hospitals or post-acute care facilities, or were scheduled for transfer to other hospitals during the index hospitalization."

**Correction 3 (Results):**

- **Original:** "A total of 2308 patients underwent tracheostomy during their index hospitalization in Japan between April 2016 and March 2019 in 219 hospitals (Fig. 1)."
- **Revised:** "A total of 2308 patients underwent tracheostomy during their index hospitalization across 219 hospitals (Fig. 1)."

**Correction 4 (Fig. 2):**

- Original figure:
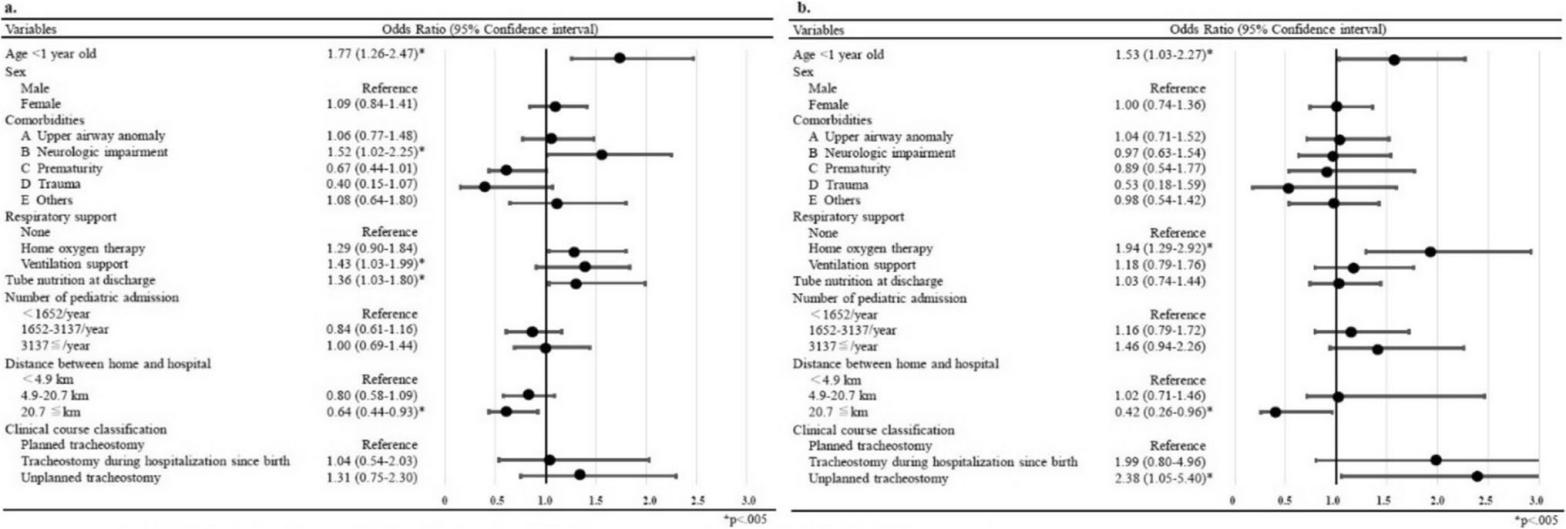
- Revised figure:

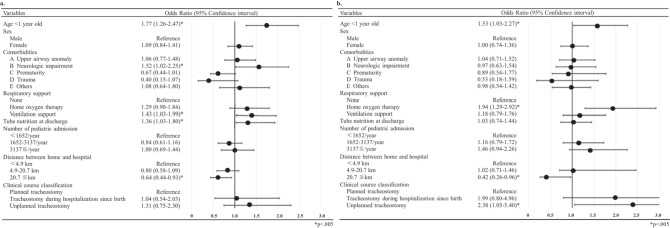

The original article has been corrected.

