# Supplementary material for: Risk factors associated with unplanned readmissions and frequent out-of-hour emergency department visits after pediatric tracheostomy: a nationwide inpatient database study in Japan
Source: Eur J Pediatr. 2025 Jun 17;184(7):422. doi: 10.1007/s00431-025-06242-1 (PMC12170732; doi:10.1007/s00431-025-06242-1)
Supplement: Supplementary file 1 — Supplementary file1 (DOCX 18.2 KB) [file 431_2025_6242_MOESM1_ESM.docx]

**Online resource 1: Comorbidity Categories, Specific Diseases and ICD-10 Diagnostic Codes**

| **Upper airway anormaly** | **ICD-10 Diagnostic Codes** |
| --- | --- |
| Stenosis and obstruction of upper respiratory tract | J988, J398, J399, J392, J386 |
| Congenital malformations of mandible and mandibulofacial dysostosis | Q18.5, Q18.7-9, Q75.4 |

| **Neurological Impairment Categories** | **ICD-10 Diagnostic Codes** |
| --- | --- |
| Diseases of the nervous system | G00-99, P90, P91, P94 |
| Viral infections of the central nervous system | A80-89 |
| Sequelae of nerve system tuberculosis, poliomyelitis, and leprosy | B900, B91, B94 |
| Benign neoplasm of brain and other parts of central nervous system | D33 |
| Congenital malformations of the nervous system | Q00-04, Q05.0-4, Q07 |
| Genetic Conditions | Q90-93, Q95, Q97-99 |
| Certain conditions originating in the perinatal period | P10, P11, P20, P21, P52 |

| **Prematurity** | **ICD-10 Diagnostic Codes** |
| --- | --- |
| Disorders related to short gestation and low birth weight | P07, P05 |

| **Trauma** | **ICD-10 Diagnostic Codes** |
| --- | --- |
| Injuries to the head and neck | S0, S1, S17, S18, S197-199, T90, T91. T950, S02, S04, S06 |
| Injuries of spine and spinal cord | T06-T062, T093,T094, T093, T094, T08, |
